# Characterization of a novel two-component Na^+^(Li^+^, K^+^)/H^+^ antiporter from *Halomonas zhaodongensis*

**DOI:** 10.1038/s41598-017-04236-0

**Published:** 2017-06-26

**Authors:** Lin Meng, Fankui Meng, Rui Zhang, Zhenglai Zhang, Ping Dong, Kaifu Sun, Jin Chen, Wei Zhang, Mingxue Yan, Jincheng Li, Heba Abdel-motaal, Juquan Jiang

**Affiliations:** Department of Microbiology and Biotechnology, College of Life Sciences, Northeast Agricultural University, Harbin, China

## Abstract

In this study, genomic DNA was screened for novel Na^+^/H^+^ antiporter genes from *Halomonas zhaodongensis* by selection in *Escherichia coli* KNabc lacking three major Na^+^/H^+^ antiporters. Co-expression of two genes designated *umpAB*, encoding paired homologous unknown membrane proteins belonging to DUF1538 (domain of unknown function with No. 1538) family, were found to confer *E. coli* KNabc the tolerance to 0.4 M NaCl and 30 mM LiCl, and an alkaline pH resistance at 8.0. Western blot and co-immunoprecipitation establish that UmpAB localize as a hetero-dimer in the cytoplasmic membranes. Functional analysis reveals that UmpAB exhibit pH-dependent Na^+^(Li^+^, K^+^)/H^+^ antiport activity at a wide pH range of 6.5 to 9.5 with an optimal pH at 9.0. Neither UmpA nor UmpB showed homology with known single-gene or multi-gene Na^+^/H^+^ antiporters, or such proteins as ChaA, MdfA, TetA(L), Nap and PsmrAB with Na^+^/H^+^ antiport activity. Phylogenetic analysis confirms that UmpAB should belong to DUF1538 family, which are significantly distant with the above-mentioned proteins with Na^+^/H^+^ antiport activity. Taken together, we propose that UmpAB represent a novel two-component Na^+^(Li^+^, K^+^)/H^+^ antiporter. To the best of our knowledge, this is the first report on the functional analysis of unknown membrane proteins belonging to DUF1538 family.

## Introduction

In prokaryotes, Na^+^/H^+^ antiporters are ubiquitous secondary transporters that catalyze the efflux of intracellular alkali cations such as Na^+^, Li^+^ or K^+^ in exchange for external protons, which play a vital role in reducing the cytoplasmic concentration of toxic alkali cations and supporting Na^+^(K^+^)-dependent intracellular pH homeostasis under alkaline conditions^1–4^. They were also designated Na^+^(Li^+^)/H^+^ antiporters, due to Na^+^/H^+^ antiport activity together with Li^+^/H^+^ antiport activity. Some of them were sometimes reported to exhibit K^+^/H^+^ antiport activity^2–4^. Since the gene *ant* from *Escherichia coli* was found for the first time to affect the Na^+^/H^+^ antiporter activity of the host and therefore designated *nhaA* ^5^, Na^+^(Li^+^)/H^+^ antiporter genes or the genes with Na^+^(Li^+^)/H^+^ antiport activity have been increasingly cloned and functionally identified in *E*. *coli* mutants EP432^6^ or KNabc^7^, which lack two or three major antiporters. Combined on the number of encoding genes with the specificity for the substrates, Na^+^/H^+^ antiporters are divided into two major categories: one category of Na^+^/H^+^ antiporters mainly with the substrates for Na^+^ and Li^+^ are encoded by a single gene such as *nhaA* ^5, 8^, *nhaB* ^9, 10^, *nhaC* ^11^, *nhaD* ^12–16^, *nheE* ^17^, *napA* ^18^, *nhaP* ^19^, *nhaG* ^20^ or *nhaH* ^21^, all of which are grouped into the monovalent Cation/Proton Antiporter 1 (CPA-1) family with the exception of NapA sharing the high identity with K^+^/H^+^ antiporters that are grouped into the CPA-2 family^22^. The other category of monovalent cation/proton antiporters with the substrates for Na^+^ and Li^+^, and sometimes for K^+^, are encoded by a multi-cistronic operon containing six or seven genes with different designations such as *mrp* ^23–25^, *mnh* ^26^, *pha* ^27–30^ or *sha* ^31^, which are grouped into the CPA-3 family due to its distinctive multi-gene structural properties^22^. In addition to two above-mentioned major categories, some non-specific Na^+^/H^+^ antiporters were also continually shown to exhibit Na^+^/H^+^ antiport activity. For example, ChaA classified into CPA-1 family was reported to have properties of Ca^2+^(Na^+^)/H^+^ antiporter^32^ and K^+^/H^+^ antiporter^33^. MleN, a novel HCT (2-hydroxy-carboxylate transporter) family transporter, was identified to exhibit Na^+^/H^+^ antiport activity coupled with the exchange of periplasmic malate with cytoplasmic lactate^34^. An unique tetracycline/H^+^ antiporter, TetA(L), belonging to MF (major facilitator) family, and a primary Na^+^ pump, Nap, belonging to NDH (NADH dehydrogenase) family were reported to possess Na^+^/H^+^ antiport activity^35, 36^. An *E*. *coli* multidrug resistance (MDR) protein, MdfA, belonging to MF family with a broad-specificity MDR phenotype^37^ was also characterized to display Na^+^(K^+^)/H^+^ antiport activity^38^. Putative paired small multidrug resistance (PSMR) family proteins, PsmrAB, as a homolog of YvdSR, were characterized to function mainly as a novel two-component Na^+^/H^+^ antiporter^39^.

In our previous study, strain NEAU-ST10-25^T^ isolated from Na_2_CO_3_-type saline-alkaline soils in Zhaodong City, Heilongjiang Province, China, has been identified to represent a novel species of the genus *Halomonas, Halomonas zhaodongensis* ^40^. This novel strain is a moderately halophilic and alkaliphilic bacterium with the growth range of 0–2.5 M NaCl (optimum 0.5 M) and pH 6–12 (optimum pH 9.0), and thus could have developed sophisticated mechanisms to maintain its intracellular steady osmotic and ionic states. Since almost all halophilic microorganisms have the ability to expel Na^+^ from the interior of the cells using Na^+^/H^+^ antiporters^41, 42^, it is very likely that a variety of important Na^+^/H^+^ antiporters, or even novel proteins which have not been reported to possess Na^+^/H^+^ antiport activity, exist in this novel strain, NEAU-ST10-25^T^, a moderate halophile and alkaliphile which can tolerate up to 2.5 M NaCl and pH 12.

To obtain as many (especially novel) genes with Na^+^/H^+^ antiport activity as possible, genomic DNA was screened from strain NEAU-ST10-25^T^ by selection in *E*. *coli* KNabc lacking three major Na^+^(Li^+^)/H^+^ antiporters. Of several resultant genes, one Group 1 *mrp* operon has been identified to encode a novel monovalent cation/proton antiporter^25^. In contrast, other genes were predicted to encode proteins that have not been reported to possess Na^+^(Li^+^)/H^+^ antiport activity as yet. However, they were found to exhibit Na^+^(Li^+^)/H^+^ antiport activity and some of them also possess K^+^/H^+^ antiport activity. Among these non-specific Na^+^(Li^+^)/H^+^ antiporter genes, two genes designated *umpAB* were found to encode paired unknown homologous membrane proteins belonging to DUF1538 family, and the sole co-expression of them could confer *E*. *coli* KNabc the tolerance to 0.4 M NaCl and 30 mM LiCl, and an alkaline pH resistance at 8.0. In this study, we reported the cloning and functional analysis of *umpAB* and finally propose that UmpAB should function as a novel two-component Na^+^(Li^+^, K^+^)/H^+^ antiporter.

## Results

### Cloning and sequence analysis of Na^+^/H^+^ antiporter genes

**Figure 1:**
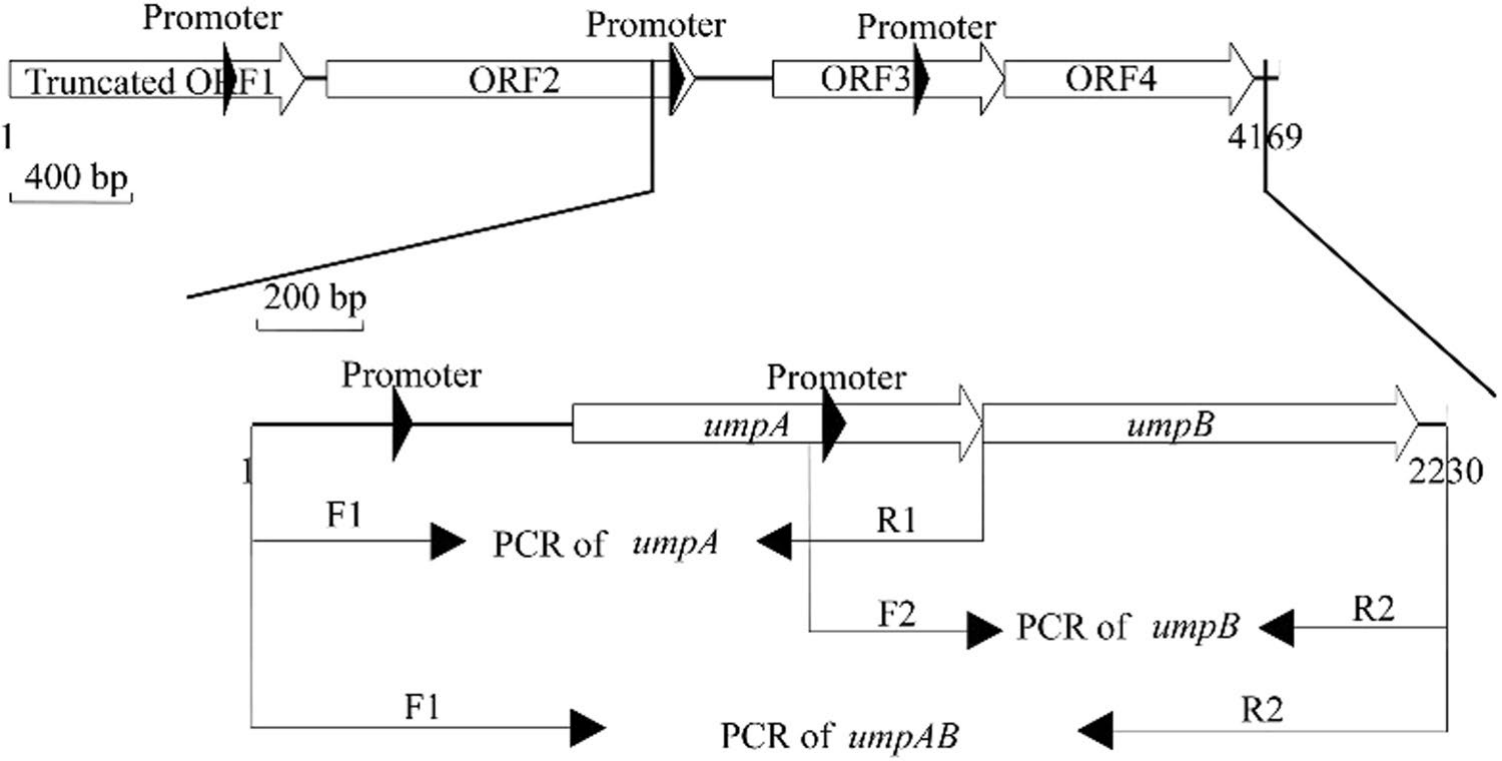
The mapping of the inserted DNA fragment in the recombinant plasmid pUC-ZD-6 and subcloning strategy of *umpA* gene, *umpB* gene or both. One 5′-end truncated ORF (ORF1) and three intact ORFs (ORF2-4) are included in this DNA fragment. 5′-end truncated ORF1 begins from No. 1 bp of this 4.2-kb DNA fragment. Each of ORF2-4 is preceded by a respective promoter-like sequence and a respective Shine-Dalgarno (SD) sequence. The open arrow stands for ORF and filled one for the predicted promoter. Subcloning of *umpA* gene, *umpB* gene or both including their respective promoter-like and SD sequences was carried out by PCR amplification, purification and re-ligation into a cloning vector pUC18. The line arrows stand for the primers for subcloning of the different ORFs by PCR.

**Figure 2:**
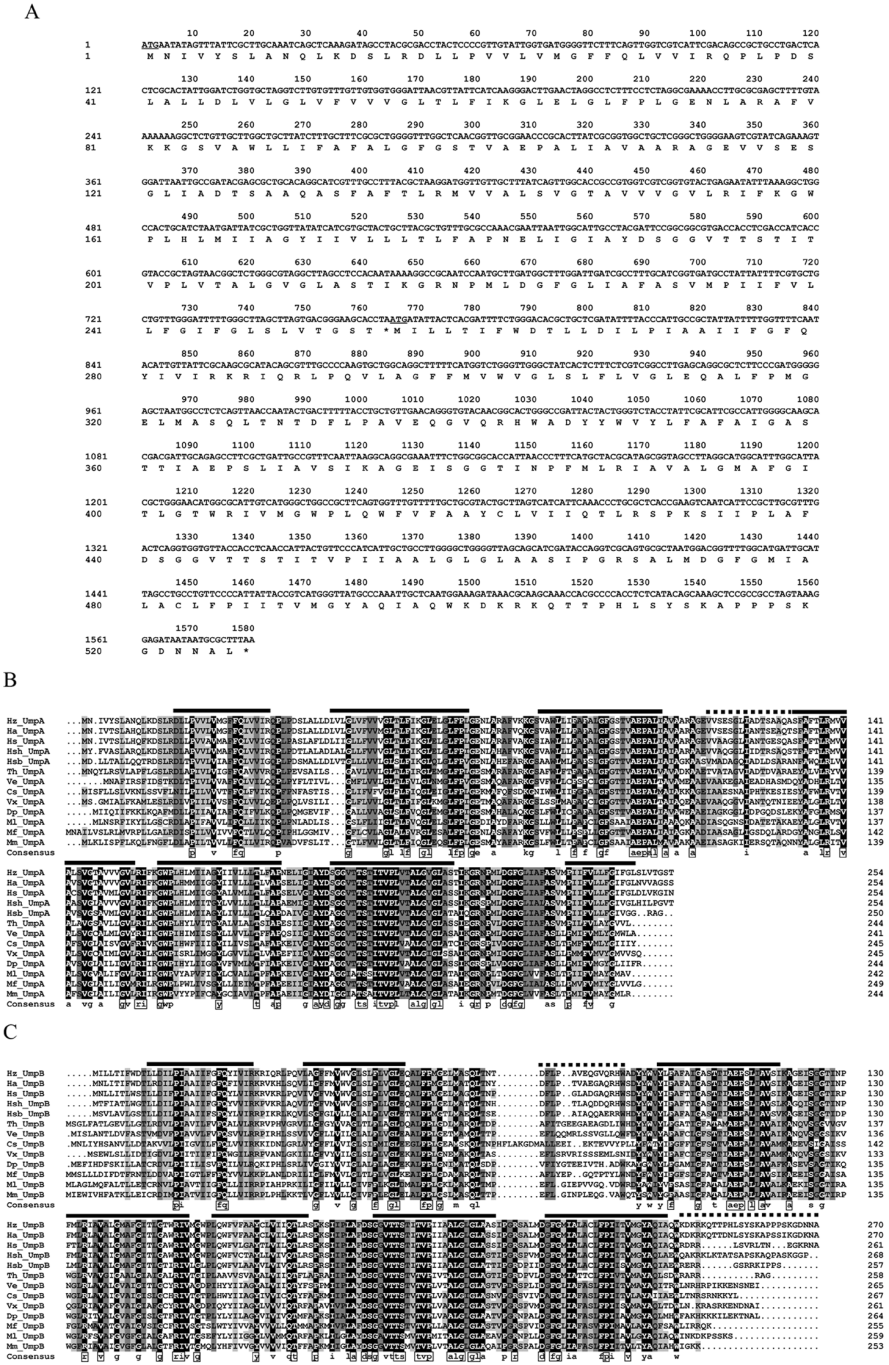
The nucleotide sequences and the deduced amino acid sequences of UmpAB and alignment of UmpAB with its most closely related holomogs clustered within the neighbour-joining phylogenetic tree. (**A**) The nucleotide sequences and the deduced amino acid sequences of UmpAB. Initial codons of UmpA and UmpB are underlined and stop codons are indicated by the asterisks, respectively. (**B** and **C**) Alignment between UmpAB and their respective homologs of DUF1538 family protein pairs. The 12 homologs at a range of the respective identities of 54% to 93% for UmpA and 52% to 90% for UmpB were selected, which clustered with either UmpA or UmpB within the neighbour-joining phylogenetic tree with the respective bootstrap values of 96% and 99%. Accession.version numbers and the hosts of selected UmpAB homologs are shown in the neighbour-joining phylogenetic tree in Fig. 6 and Table S1. Shading homology corresponds to 100% (black), >75% (grey), ≥50% (lightgrey) and <50% (white) amino acid identity, respectively. The seven putative transmembrane segments are marked with bold solid lines above the alignment. The additional parts of Loop III-IV for UmpA, Loop II-III for UmpB and the hydrophilic C terminus for UmpB are marked with bold dotted lines above the alignment. The highly conserved residues between UmpAB homologs are highlighted with the open rectangles in the consensus sequence.

For the cloning of Na^+^/H^+^ antiporter genes, *Sau*3AI-digested genomic DNA from strain NEAU-ST10-25^T^ was electroporated into *E. coli* KNabc and its transformants were screened on the LBK medium plates containing 0.2 M NaCl. Of several resultant clones, one recombinant plasmid designated pUC-ZD-6 [pUC18 carrying a 4.2-kb DNA fragment (Fig. 1)] enabled *E*. *coli* KNabc to grow on the LBK medium plate containing 0.2 M NaCl, which is the upper limit of NaCl tolerance concentrations for *E. coli* KNabc lacking three major Na^+^/H^+^ antiporters (*nhaA*::Km^R^, *nhaB*::Em^R^, *chaA*::Cm^R^)^7^. Sequence analysis showed that one 5′-end truncated ORF (ORF1) and three intact ORFs (ORF2-4) are included in this 4.2-kb DNA fragment (Fig. 1). With the exception of 5′-end truncated ORF1 beginning from No. 1 bp of this 4.2-kb DNA fragment, ORF2-4 are preceded by a respective promoter-like sequence and a respective Shine-Dalgarno (SD) sequence. Also, the stop codon (TAA) of ORF3 overlaps at the site of base “A” with the initial codon (ATG) of ORF4 (Fig. 2A). 5′-end truncated ORF1 has the highest identity of 90% with the amino acid sequence from No. 142 residue to No. 464 residue of a putative citrate transporter consisting of 464 residues (accession.version No. EGP18268.1) from *Halomonas* sp. TD01, ORF2 has the highest identity of 91% with a putative arabinose efflux permease (accession.version No. SEO09378.1) belonging to MF family from *H. aquamarina*, ORF3-4 have the respective highest identity of 93% and 90% with paired unknown homologous membrane proteins (accession.version No. WP_030072713.1/WP_030072711.1) belonging to DUF1538 family from *H. alkaliantarctica* (Fig. 2B and C, Table S1).

Among the above-mentioned four ORFs, ORF1 preceded by no promoter or SD sequences is incomplete and therefore can’t restore the growth of *E. coli* KNabc in the presence of 0.2 M NaCl. As for the other three intact ORFs, ORF2 was predicted to be a membrane protein composed of ten transmembrane segments (TMSs) with a calculated molecular weight of 42, 630.6 Dalton and a pI of 9.37. Among the 402 deduced amino acid residues of ORF2, 239 residues are hydrophobic, indicating that ORF2 is of low polarity. ORF3 was predicted to be a membrane protein consisting of seven TMSs including TMS I (17–34), TMS II (46–71), TMS III (83–108), TMS IV (128–153), TMS V (165–182), TMS VI (194–214), TMS VII (226–251) (Fig. 2B). The deduced amino acid sequence of ORF3 consists of 254 residues (Fig. 2A) with a calculated molecular weight of 26, 570.9 Dalton and a pI of 7.53. Among the 254 residues of ORF3, 188 residues are hydrophobic, indicating that ORF3 is of low polarity. ORF4 was also predicted to be a membrane protein consisting seven TMSs including TMS I (11–30), TMS II (40–58), TMS III (92–115), TMS IV (125–148), TMS V (152–172), TMS VI (188–212), TMS VII (216–236) (Fig. 2C). The deduced amino acid sequence of ORF3 consists of 271 residues (Fig. 2A) with a calculated molecular weight of 29, 555.8 Dalton and a pI of 9.37. Among the 271 residues of ORF3, 189 residues are hydrophobic, indicating that ORF4 is of low polarity. Based on the above sequence analysis, each of ORF2-4 is very likely to possess Na^+^/H^+^ antiport activity, since Na^+^/H^+^ antiporters must be membrane proteins of low polarity^1–4^.

### Identification of ORFs with Na^+^/H^+^ antiport activity

**Figure 3:**
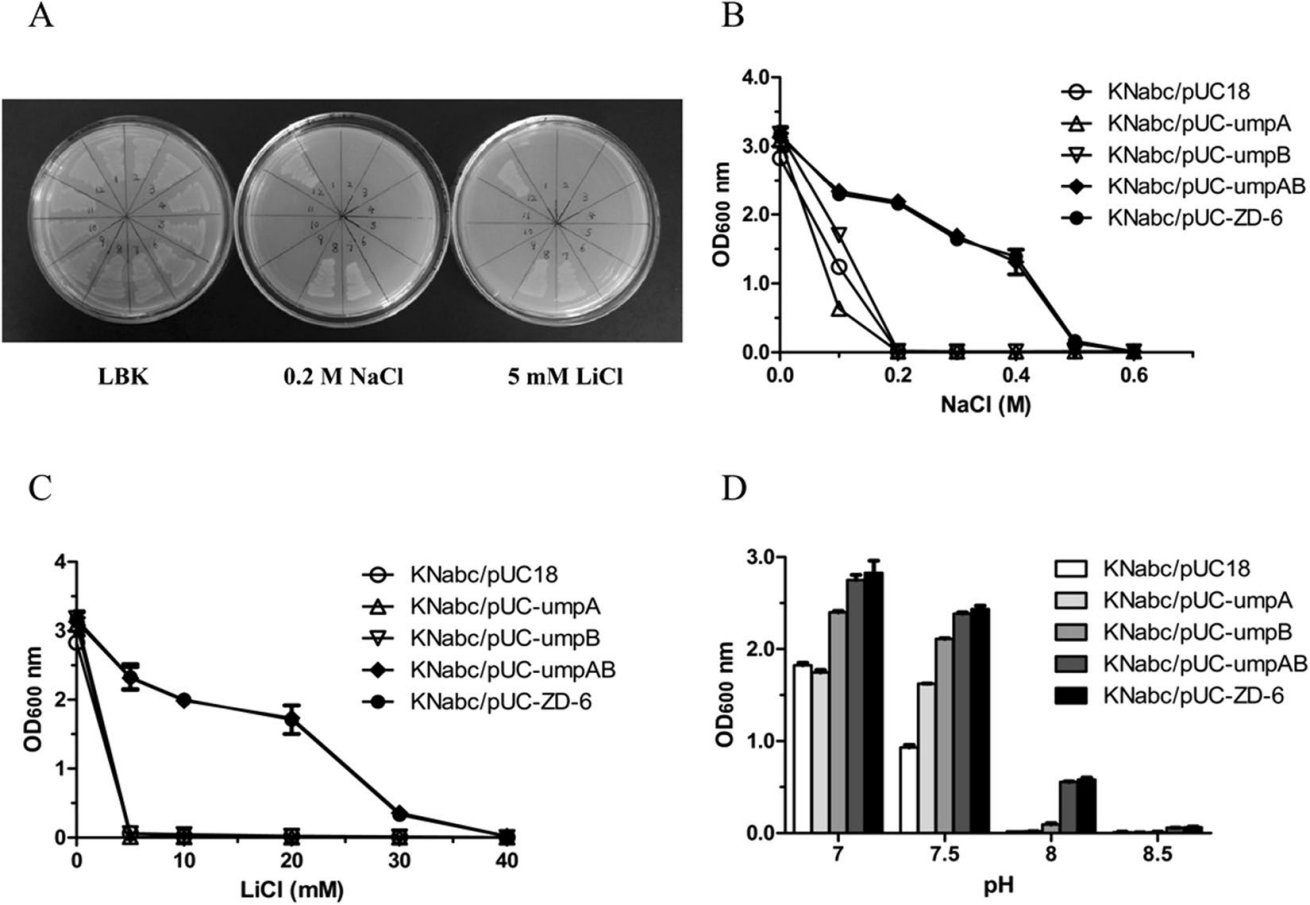
Salt tolerance and alkaline pH resistance of *E. coli* KNabc. For the complementation test (**A**), *E. coli* KNabc transformant cells were grown on the LBK medium plates at pH 7.0 containing no addition of NaCl or LiCl, 0.2 M NaCl or 5 mM LiCl. (1) KNabc/pET19, (2) KNabc/pET19-truncated ORF1, (3) KNabc/pUC18, (4) KNabc/pUC-ORF2, (5) KNabc/pUC-umpA, (6) KNabc/pUC-umpB, (7) KNabc/pUC-umpAB, (8) KNabc/pUC-ZD-6, (9) KNabc/pETDuet-1, (10) KNabc/pETDuet-1-umpA, (11) KNabc/pETDuet-1-umpB, (12) KNabc/pETDuet-1-umpAB. For the salt tolerance test, 1% overnight cultures of *E. coli* KNabc transformant cells were grown in the LBK medium at pH 7.0 containing 0–0.6 M NaCl (**B**), or 0–40 mM LiCl (**C**), followed by incubation at 37 °C. To test the effect of pH on cell growth (**D**), 1% overnight cultures of *E. coli* KNabc transformant cells were innoculated into fresh LBK medium plus 50 mM NaCl at indicated pH values (7.0–8.5) by adding the Hepes-Tris buffer (final concentration at 100 mM), followed by incubation at 37 °C. The above-mentioned cell growth was ended after 24 h and monitored turbidimetrically at 600 nm. Each data point represents the average of three independent determinations.

To identify the exact ORF(s) with Na^+^/H^+^ antiport activity, each of ORF2-4 with its respective promoter-like and SD sequence was subcloned by PCR into a cloning vector, pUC18. To avoid the inactivity of their original promoters in *E. coli* KNabc, each of ORF2-4 was separately inserted just downstream from the *lac* promoter of pUC18 in the forward orientation. The strategy of subcloning of ORF3, ORF4 or both was carried out as shown in Fig. 1. The strategy of subcloning of ORF2 is similar to that of ORF3 or ORF4. Even though it’s impossible that 5′-end truncated ORF1 preceded by no promoter or SD sequences can be transcribed and translated in *E. coli*, it was also fused in frame with an N-terminal His_6_ tag in an expression vector, pET19 (Novagen Ltd., USA). Each subclone was transformed separately into *E. coli* KNabc for the complementation growth tests. The results showed that no single ORF could enable *E. coli* KNabc to grow on the LBK medium plates containing 0.2 M NaCl or 5 mM LiCl (Fig. 3A). Considering that ORF3 and ORF4 exist in pairs (Fig. 2A), and both of them are highly homologous membrane proteins belonging to DUF1538 family (Fig. 2B), it’s very likely that ORF3-4 can exhibit Na^+^/H^+^ antiport activity only if they are co-expressed as a hetero-dimer. Therefore, both of them were subcloned together into pUC18 and found to exactly succeed in restoring the growth of *E. coli* KNabc on the LBK medium plates containing 0.2 M NaCl or 5 mM LiCl (Fig. 3A). It should be stressed that ORF3-4 with their respective predicted promoters were inserted just downstream from the *lac* promoter of pUC18 in the opposite orientation in the recombinant plasmid pUC-ZD-6. Therefore, it is concluded that the original promoters of ORF3-4 should be functional in the *E*. *coli* cells. In order to describe the following identification, ORF3- and ORF4-encoded genes were designated *umpA* and *umpB*, respectively, based on the identity with unknown membrane proteins belonging to DUF1538 family. The resultant plasmids containing subcloned *umpA*, *umpB* or both with their respective promoter-like and SD sequences were therefore designated pUC-umpA, pUC-umpB and pUC-umpAB, respectively. To confirm whether the sole co-expression of *umpA* and *umpB* could rescue *E. coli* KNabc, *umpA* and *umpB* genes were constructed into a co-expression vector, pETDuet-1 (Novagen Ltd., USA). In the construct designated pETDuet-1-umpAB, UmpA was fused in frame with an N-terminal His_6_ tag in the multiple cloning site 1 (MCS1) and UmpB followed by an in-frame c-Myc tag and a stop codon was inserted into the multiple cloning site 2 (MCS2). Also, the sole expression vectors of *umpA* or *umpB* genes designated pETDuet-1-umpA and pETDuet-1-umpB, respectively, were also constructed by only fusing UmpA with an N-terminal His_6_ tag in MCS1 of pETDuet-1 or only inserting UmpB followed by the in-frame c-Myc tag and a stop codon into MCS2 of pETDuet-1. Sequencing analysis revealed that UmpA succeeded in being fused in frame with an N-terminal His_6_ tag and/or UmpB was also fused with the c-Myc tag followed by a stop codon. The complementation growth tests showed that *E*. *coli* KNabc/pETDuet-1-umpAB, but KNabc/pETDuet-1 (as a negative control), KNabc/pETDuet-1-umpA or KNabc/pETDuet-1-umpB not, could grow on the LBK medium plates containing 0.2 M NaCl or 5 mM LiCl (Fig. 3A).

### Detailed growth test for salt tolerance and alkaline pH resistance of UmpAB

To test the ability of UmpAB to induce salt tolerance, *E. coli* KNabc/pUC-umpAB, KNabc/pUC-umpA and KNabc/pUC-umpB, KNabc/pUC-ZD-6 as a positive control and KNabc/pUC18 as a negative control were grown in the LBK medium containing 0–0.6 M NaCl or 0–40 mM LiCl. As shown in Fig. 3B and C, *E. coli* KNabc/pUC-ZD-6 and KNabc/pUC-umpAB could grow in the presence of 0.4 M NaCl or 30 mM LiCl, but none of KNabc/pUC-umpA, KNabc/pUC-umpB or KNabc/pUC18 could grow in the presence of 0.2 M NaCl or 5 mM LiCl. To analyze the resistance of UmpAB to alkaline pH, all the above-mentioned *E*. *coli* KNabc transformants were also grown in the LBK medium at the pH values from 7.0 to 8.5. As shown in Fig. 3D, the growth of KNabc/pUC-umpA, KNabc/pUC-umpB and KNabc/pUC18 were greatly reduced under alkaline conditions, especially inhibited at pH 8.0, compared with that below neutral pH, whereas co-expression of *umpAB* conferred *E. coli* KNabc the capability, similar to that of the original clone in the recombinant plasmid pUC-ZD-6, to grow under alkaline conditions. Moreover, compared with that of KNabc/pUC18, the growth of KNabc/pUC-umpB were significantly better in the presence of 0.1 M NaCl (Fig. 3B) whereas that of KNabc/pUC-umpA was significantly inhibited under the same condition (Fig. 3B). Also, either KNabc/pUC-umpA or KNabc/pUC-umpB showed the higher alkaline resistance at pH 7.5, compared with KNabc/pUC18 (Fig. 3D). Therefore, it is concluded that separately-subcloned *umpA* or *umpB* should be expressed in the *E*. *coli* KNabc cells. In order to confirm NaCl tolerance or alkaline pH resistance of either UmpA or UmpB, the above-mentioned *E*. *coli* KNabc transformants were grown in the LBK medium containing NaCl concentrations varied from 0 to 150 mM at the pH values from 7.0 to 8.5. As shown in Fig. S1, the sole expression of either *umpA* or *umpB*, especially the latter, could offer *E. coli* KNabc NaCl tolerance or alkaline pH resistance, to some extent.

### Western blot detection and localization of UmpA and UmpB

**Figure 4:**
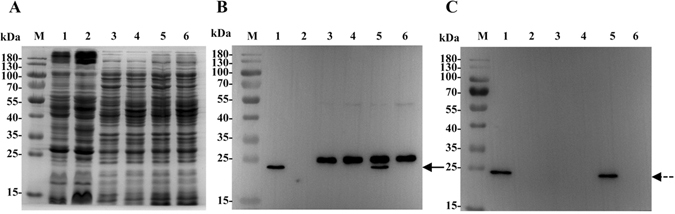
Western blot detection and localization of UmpA and UmpB. For the detection and localization of UmpA and UmpB, *E*. *coli* KNabc cells carrying pETDuet-1-umpAB and the empty vector pETDuet-1 (as a negative control) were grown in the LBK medium to OD_600 nm_ between 0.4 and 0.6 at 37 °C, followed by induction by the addition of isopropyl-β-D-thiogalactoside to a final concentration of 1 mM at 28 °C for an additional 6 h and then harvested by centrifugation at 5, 000 g, 4 °C for 10 min and washed three times with the 10 mM Tris-HCl buffer (pH 7.5). The membrane fraction, cytoplasmic ones and cell extract from *E. coli* KNabc/pETDuet-1-umpAB (Lanes 1, 3, 5) and KNabc/pETDuet-1 (Lanes 2, 4, 6) were sampled, respectively, and then used for SDS-PAGE (**A**) and western blots. The positions of target proteins, UmpA (**B**) fused with a N-terminal His_6_ tag and UmpB (**C**) fused with a C-terminal c-Myc tag, are shown with a solid line arrow and a dotted line arrow, respectively.

For the detection and localization of UmpA and UmpB, *E. coli* KNabc with pETDuet-1-umpAB or pETDuet-1 (as a negative control) were used for the preparation of the samples for cell extract, membrane fraction and cytoplasmic fraction. As shown in Fig. 4, the expression of both UmpA and UmpB was detected in the cell extract and membrane fraction from the cells of *E. coli* KNabc/pETDuet-1-umpAB, but not in those from KNabc/pETDuet-1. It should be pointed out that a non-specific protein with a molecular weight of around 25 kDa was detected in the cell extract and cytoplasmic fraction from the cells of both *E. coli* KNabc/pETDuet-1-umpAB and KNabc/pETDuet-1, but not from membrane fraction from the cells of *E. coli* KNabc/pETDuet-1-umpAB or KNabc/pETDuet-1 (Fig. 4B). This result reveals that this non-specific protein should be localized in the cytoplasm of *E. coli* cells. Also, the anticipated UmpA with a molecular weight of obviously lower than 25 kDa was solely detected in the cell extract and membrane fraction from the cells of *E. coli* KNabc/pETDuet-1-umpAB, but not those from KNabc/pETDuet-1 (Fig. 4B), which established that it is indeed the target protein for UmpA. More importantly, the anticipated UmpB, which is longer than UmpA, was also detected at the position of obviously lower than 25 kDa (Fig. 4C), which further excluded the possibility of the above-mentioned non-specific protein being the target protein.

### Determination of UmpAB as a hetero-dimer by co-immunoprecipitation

**Figure 5:**
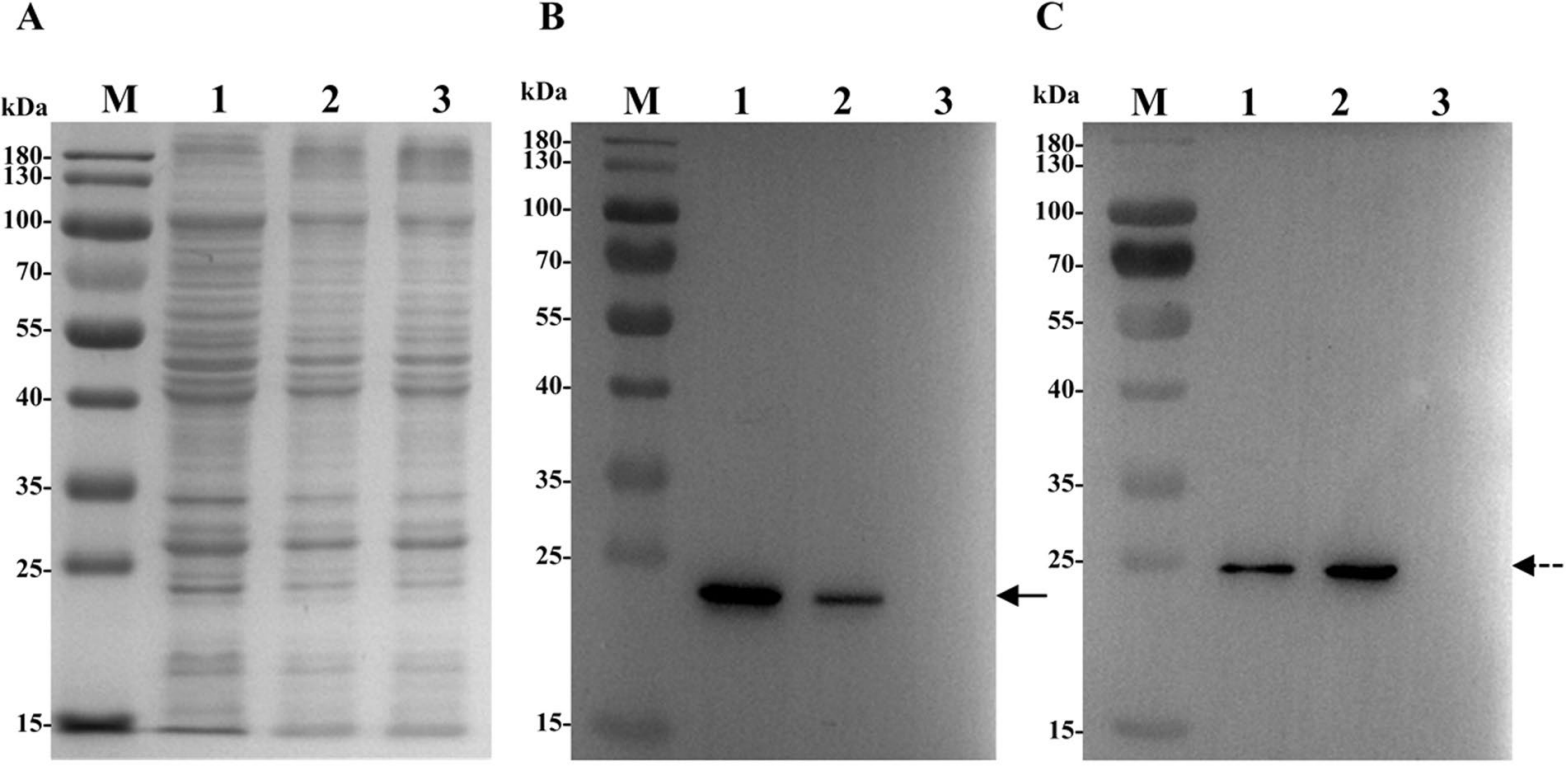
Determination of UmpA and UmpB as a hetero-dimer by co-immunoprecipitation. For the determination of UmpA and UmpB as a hetero-dimer, the stored membrane fraction containing 1.2 mg total membrane protein from *E. coli* KNabc cells carrying pETDuet-1-umpAB were re-suspended in an ice-cold commercially-available buffer containing 20 mM Tris-HCl (pH 7.5), 150 mM NaCl, 1% Triton X-100 and a certain amount of protease inhibitors including sodium pyrophosphate, β-glycerophosphate, EDTA, sodium ortovanadate and leupeptin. And then the primary antibody [a rabbit anti-His_6_-tag antibody (Abcom Ltd, China) for UmpA] was added to the solubilized membrane proteins and precipitated with protein agarose A-sepharose. After washing three times with the ice-cold above-mentioned commercially-available buffer, the precipitates (Lane 2), together with membrane fraction (as a positive control, Lane 1) and the rabbit IgG mixed with the solubilized membrane proteins by the protein agarose A-sepharose (as a negative control, Lane 3), were used for the SDS-PAGE (**A**) and western blots. To avoid the visualization of the light chain and the heavy chain from a rabbit anti-His_6_-tag antibody in the precipitates, His_6_-tag detection (**B**) was done by using a mouse anti-His_6_-tag antibody and a goat anti-mouse horseradish peroxidase-labelled secondary antibody. c-Myc-tag detection (**C**) was done by using a mouse anti-c-Myc-tag antibody and a goat anti-mouse horseradish peroxidase-labelled secondary antibody. The positions of target proteins, UmpA fused with a N-terminal His_6_ tag and UmpB fused with a C-terminal c-Myc tag, are shown with a solid line arrow and a dotted line arrow, respectively.

For the determination of UmpA and UmpB as a hetero-dimer, co-immunoprecipitation of UmpB by UmpA was performed by using the membrane fraction from the cells of *E. coli* KNabc/pETDuet-1-umpAB. The samples for the precipitates of UmpB by UmpA, together with membrane fraction (as a positive control) and the rabbit IgG mixed with the solubilized membrane proteins by the protein agarose A-sepharose (as a negative control) were used for the SDS-PAGE (Fig. 5A). It should be pointed out that the co-precipitates of UmpB by UmpA seemed to contain all the membrane proteins from KNabc/pETDuet-1-umpAB. Also, almost the same proteins appeared in the rabbit IgG mixed with the solubilized membrane proteins by the protein agarose A-sepharose (as a negative control) (Fig. 5A). However, positive signals for both UmpA and UmpB were detected in the membrane fraction from the cells of *E. coli* KNabc/pETDuet-1-umpAB whereas no signal was detected in the precipitates of the rabbit IgG mixed with the solubilized membrane proteins by the protein agarose A-sepharose (Fig. 5B and C). This indicates that these proteins in the co-precipitates of UmpB by UmpA were resulted from the non-specific binding of them with the rabbit IgG or the protein agarose A-sepharose, but not due to the primary antibody pulling the membrane vesicles through the binding to UmpA. More importantly, in the precipitates of His_6_-tag-labelled UmpA by the protein agarose A-sepharose binding with a rabbit anti-His_6_-tag antibody, not only His_6_-tag-labelled UmpA was detected by using a mouse anti-His_6_-tag antibody and a goat anti-mouse horseradish peroxidase-labelled secondary antibody (Fig. 5B), but also c-Myc-tag-labelled UmpB was detected by using a mouse anti-c-Myc-tag antibody and a goat anti-mouse horseradish peroxidase-labelled secondary antibody (Fig. 5C). This reveals that the primary antibody binding with the protein agarose A-sepharose should bind with UmpA and then co-precipitate with UmpB indeed, and also confirms that UmpA and UmpB, as well as these non-specific binding proteins, should succeed in being solubilized in the buffer containing the detergent, Triton X-100, but not in the membrane vesicles.

### Phylogenetic analysis of UmpAB based on neighbour-joining algorithm and protein alignment with their homologs

**Figure 6:**
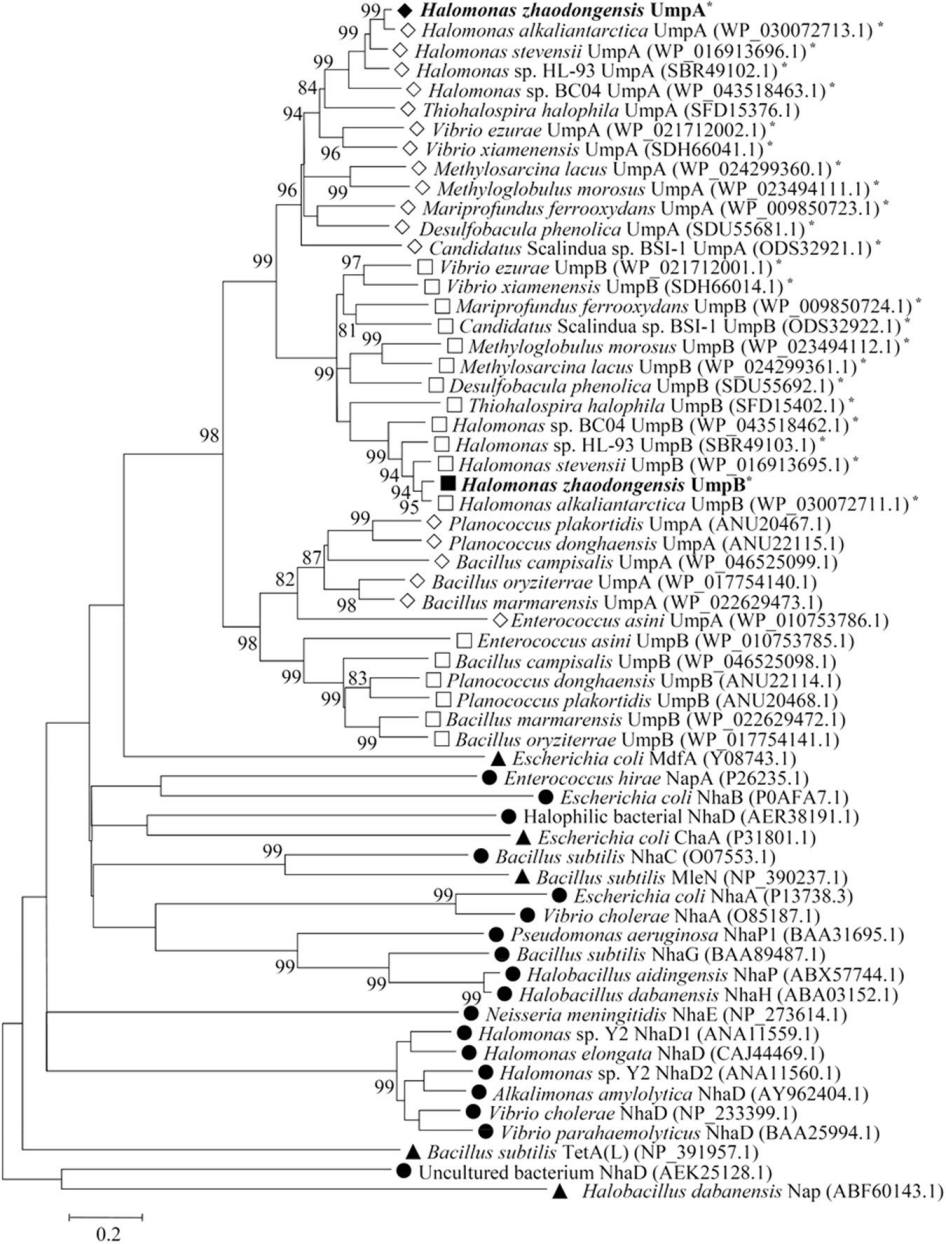
Neighbour-joining phylogenetic tree of UmpAB with their selected homologs, together with known Na^+^/H^+^ antiporters and proteins with Na^+^/H^+^ antiport activity. For the construction of phylogenetic tree, UmpA was aligned by using BLASTP at the NCBI website^43^, six closest homologs with 60–93% identities, six closer homologs with 41-59% identities, six distant homologs with 30–40% identities were downloaded (Table S1). The locations of UmpA homologs were searched in their respective genome sequences and then the corresponding UmpB homologs were selected only after they were checked to be exactly paired with UmpA ones. Also, all representatives of known single-gene Na^+^/H^+^ antiporters and other single-gene proteins with Na^+^/H^+^ antiport activity were selected. Accession.version numbers of selected proteins were shown in the parenthesis. Bootstrap values > 70% (based on 1000 replications) are shown at branch points. Bar, 0.2 substitutions per amino acid residue position. UmpAB and their respective most closely related holomogs clustered with the respective bootstrap values of 96% and 99% marked with the asterisks were aligned in Fig. 2.

Considering the result of BLASTP search using the NCBI website^43^ that both UmpA and UmpB fall within the DUF1538 family proteins, we postulated that UmpA and UmpB may belong to DUF1538 family. To show whether these two DUF1538 family proteins indeed belong to DUF1538 family and whether they share phylogenetic relationship with identified Na^+^/H^+^ antiporters and other proteins with Na^+^/H^+^ antiport activity, phylogenetic analysis based on neighbour-joining algorithm was carried out. For the construction of phylogenetic tree, UmpA was aligned by using BLASTP at the NCBI website^43^, six closest homologs with 60–93% identities, six closer homologs with 41–59% identities, six distant homologs with 30–40% identities were selected (Table S1). Because each pair of UmpA and UmpB homologs shares the identities around 50% and both of them are designated unknown membrane proteins in the protein database of NCBI website, it’s difficult to directly recognize UmpB homologs, especially below the identities of 50%, through the alignment method similar to that of UmpA homologs. Therefore, UmpB homologs were searched through the location of UmpA homologs in their respective genome sequences and then selected after they were checked to be exactly paired with UmpA homologs (Table S1). Also, all representatives of known single-gene Na^+^/H^+^ antiporters and other single-gene proteins with Na^+^/H^+^ antiport activity were selected. As shown in Fig. 6, UmpA and UmpB clustered with their respective homologs above 50% identities (Table S1) with the respective bootstrap values of 96% and 99%. Also, their homologs below 41% identities (Table S1) constitute two separate clusters with the respective bootstrap values of 82% and 98%. More importantly, UmpA and UmpB clustered with all their homologs belonging to DUF1538 family with the bootstrap value of 98%, which are significantly distant with all known Na^+^/H^+^ antiporters and proteins with Na^+^/H^+^ antiport activity (Fig. 6).

UmpA (Fig. 2B) and UmpB (Fig. 2C) were also aligned, respectively, with their 12 homologs clustered within the neighbour-joining phylogenetic tree with the respective bootstrap values of 96% and 99%. UmpA and UmpB have the respective identities of 54% to 93%, 52% to 90% (Table S1) with their corresponding homologs from *H. alkaliantarctica*, *H. stevensii*, *Halomonas* sp. HL-93, *Halomonas* sp. BC04, *Thiohalospira halophila*, *Vibrio ezurae*, *Candidatus* Scalindua sp. BSI-1, *V. xiamenensis*, *Desulfobacula phenolica*, *Methylosarcina lacus*, *Mariprofundus ferrooxydans* and *Methyloglobulus morosus*. Moreover, UmpA has the identities of 41% to 47% with UmpB homologs from the above-mentioned organisms whereas UmpB has the identities of 38% to 45% with UmpA homologs from the above-mentioned organisms (Table S1). UmpA shares the identity of 43% with UmpB within a full-length range. Also, 47 highly-conserved amino acid residues including 36 hydrophobic residues [11 glycine (G), six proline (P), five phenylalanine (F), five alanine (A), four leucine (L), three valine (V), one tyrosine (Y) and one isoleucine (I)], six charged ones [three arginine (R), two aspartic acid (D) and one glutamic acid (E)] and five polar ones [three threonine (T), one serine (S) and one glutamine (Q)] were found to commonly exist between UmpA homologs and UmpB ones (Fig. 2B and C). Of the 36 hydrophobic residues, 11 glycine residues and five phenylalanine ones are included in the UmpAB homologs (Fig. 2B and C), which is consistent with the sole information that DUF1538 family proteins contain several conserved glycine and phenylalanine residues shown at the website https://www.ncbi.nlm.nih.gov/Structure/cdd/cddsrv.cgi?ascbin=8&maxaln=10&seltype=2&uid=pfam07556. It should be stressed that either UmpA or UmpB was aligned with all known specific Na^+^(Li^+^)/H^+^ antiporters and proteins with Na^+^(Li^+^)/H^+^ antiport activity including either subunit of PsmrAB, a two-component Na^+^/H^+^ antiporter, even any subunit of multi-gene Na^+^/H^+^ antiporters, but either UmpA or UmpB showed no identity with each of them.

### Na^+^ (Li^+^, K^+^)/H^+^ antiport activity in everted membrane vesicles

**Figure 7:**
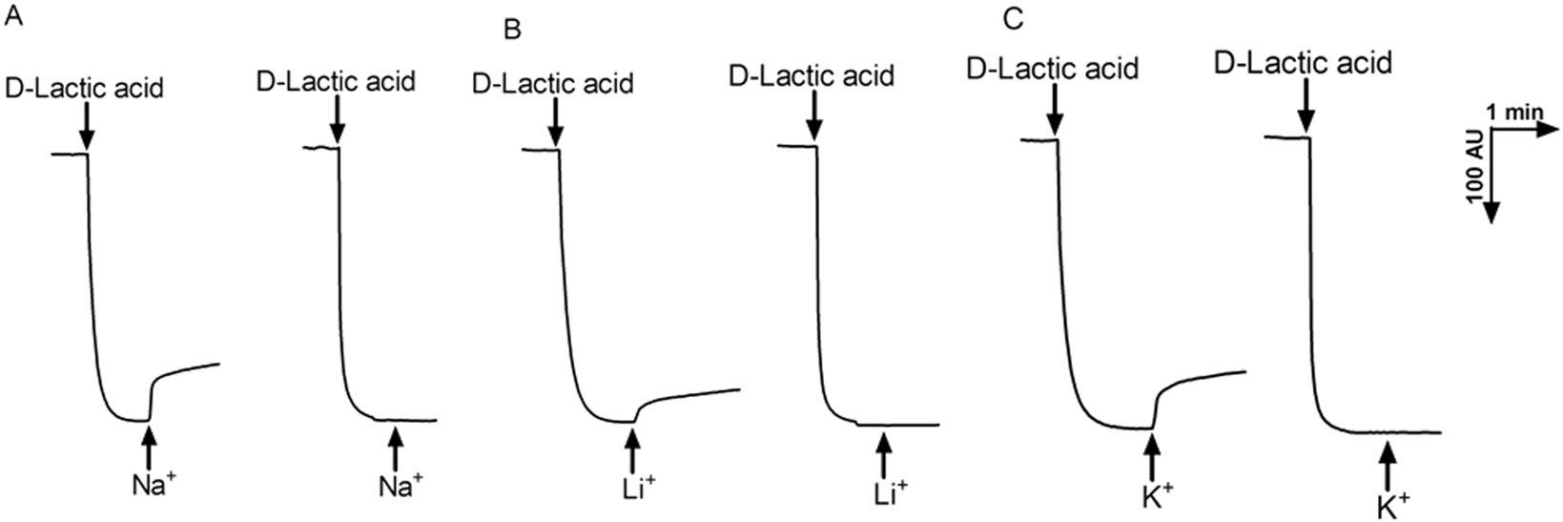
Assays for Na^+^(Li^+^, K^+^)/H^+^ antiport activity in the everted membrane vesicles. The measurements for Na^+^/H^+^ antiport (**A**), Li^+^/H^+^ antiport (**B**) and K^+^/H^+^ antiport (**C**) activity were performed in everted membrane vesicles prepared from cells of *E. coli* KNabc/pUC-umpAB (to the left) or KNabc/pUC18 (to the right) by the French pressure cell method. The highest activity at pH 9.0 were shown as the representatives of each of them. At the time points indicated by downward arrows, Tris-D-lactic acid (final concentration at 5 mM) was added to the assay mixture to initiate fluorescence quenching. At the time points indicated by upward arrows, NaCl (final concentration at 5 mM), LiCl (final concentration at 5 mM) or Na-free KCl (final concentration at 5 mM) was added to the assay mixture, respectively. Fluorescence quenching is shown in arbitrary units (AU).

**Figure 8:**
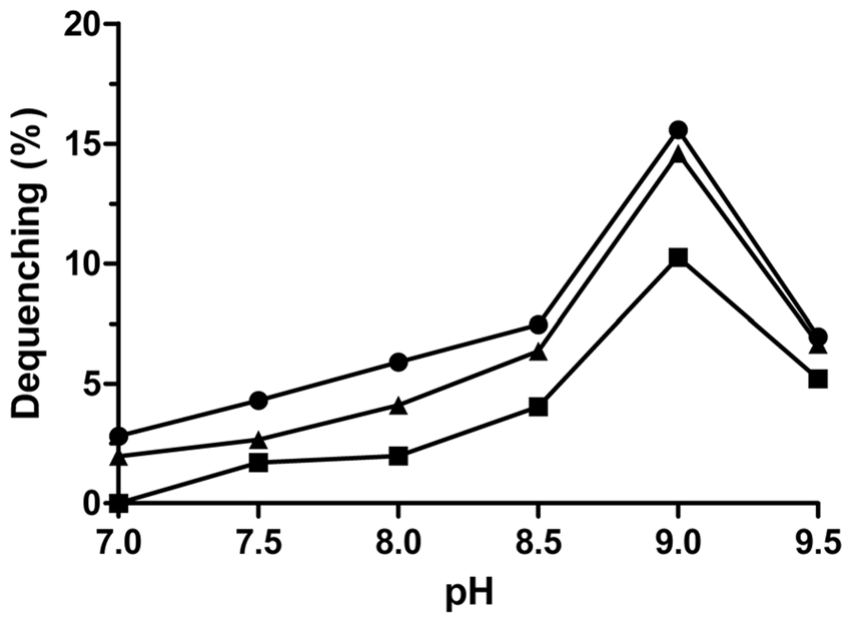
pH-dependent activity profile of UmpAB. The antiport activity was measured by the fluorescence dequenching method. Na^+^/H^+^ antiport activity (filled circle), Li^+^/H^+^ antiport activity (filled square) and K^+^/H^+^ antiport activity (filled triangle) were measured at the indicated pH values. The wavelength of excitation light was 492 nm and fluorescence was monitored at 526 nm. Each value point represents the average of three independent determinations.

Na^+^ (Li^+^, K^+^)/H^+^ antiport activity with everted membrane vesicles prepared from cells of *E*. *coli* KNabc strains carrying pUC-umpAB or pUC18 was determined by measuring the dequenching of acridine orange fluorescence upon addition of NaCl, LiCl or Na-free KCl. As shown in Fig. 7, Na^+^ (Li^+^, K^+^)/H^+^ antiport activities were detected in membrane vesicles from KNabc/pUC-umpAB, while no Na^+^/H^+^, Li^+^/H^+^ or K^+^/H^+^ antiport activity was detected in those from KNabc/pUC18. The effect of pH on Na^+^ (Li^+^, K^+^)/H^+^ antiport activity was also measured at a wide range of pH 6.5–9.5. UmpAB exhibited Na^+^/H^+^ and K^+^/H^+^ antiport activity at a wide range of pH 7.0–9.5, but Li^+^/H^+^ antiport activity at pH between 7.5 and 9.5, with an optimal for each antiport activity at pH 9.0 (Fig. 8).

### Calculation of K_0.5_ values of UmpAB for Na^+^, Li^+^ and K^+^

**Figure 9:**
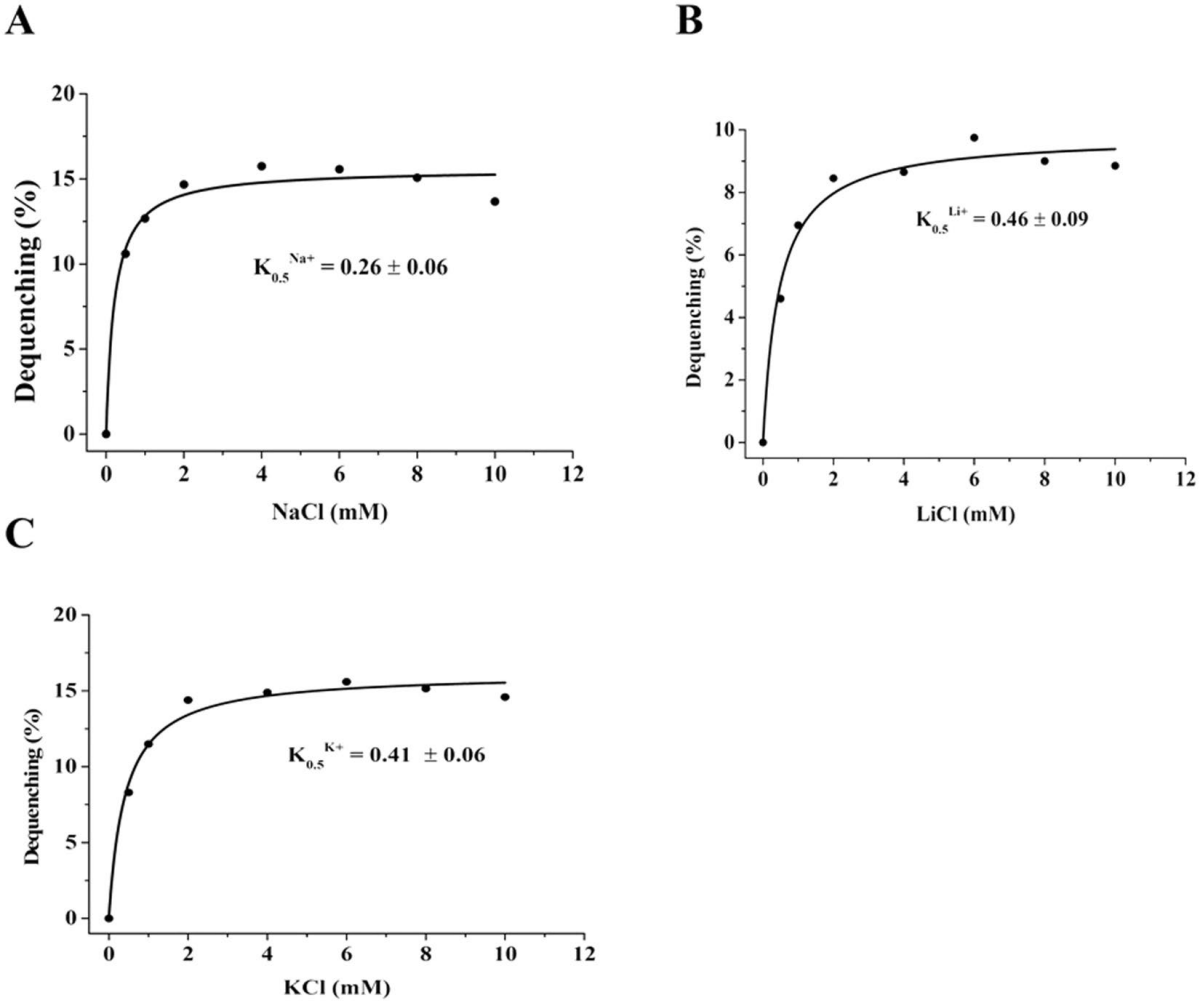
Calculation of K_0.5_ values of UmpAB for Na^+^, Li^+^ and K^+^. Na^+^/H^+^ (**A**), Li^+^/H^+^ (**B**) and K^+^/H^+^ (**C**) antiport activity of UmpAB were plotted as the respective functions of cation concentrations. K_0.5_ values of UmpAB for Na^+^, Li^+^ and K^+^ were obtained, respectively, by fitting a hyperbolic function to the data with OriginPro 8.6. Each value point represents the average of three independent determinations.

To assess the apparent affinity of UmpAB for Na^+^, Li^+^ and K^+^, the respective K_0.5_ values of UmpAB for Na^+^, Li^+^ and K^+^ were analyzed by measuring Na^+^ (Li^+^, K^+^)/H^+^ antiport activity in everted membrane vesicles from KNabc/pUC-umpAB at pH 9.0 with final concentrations of added NaCl, LiCl or Na-free KCl varied from 0 to 10 mM. K_0.5_ values of UmpAB for Na^+^, Li^+^ and K^+^ were finally calculated to be 0.26 ± 0.06 mM (Fig. 9A), 0.46 ± 0.09 mM (Fig. 9B) and 0.41 ± 0.06 mM (Fig. 9C), respectively, revealing that the preference of UmpAB for the transported monovalent cations was Na^+^ > K^+^ > Li^+^.

## Discussion

In this study, we showed that UmpAB, paired homologous DUF1538 family membrane proteins with unknown function, from the moderate halophile and alkaliphile NEAU-ST10-25^T^ function as a novel two-component Na^+^(Li^+^, K^+^)/H^+^ antiporter, which are significantly different from all known single-gene Na^+^/H^+^ antiporters^5, 8–21^, multi-gene Na^+^/H^+^ antiporters^23–31^ or proteins with Na^+^/H^+^ antiport acitivty^32–39^. The function of any member of DUF1538 family has not been experimentally characterized as yet. To the best of our knowledge, this is the first report on the functional analysis of a pair of unknown homologous membrane proteins as a representative of DUF1538 family protein pairs.

Both UmpA and UmpB were predicted to be membrane proteins consisting of seven putative TMSs, respectively (Fig. 2B and C), which was confirmed by the western blot result that both UmpA and UmpB were localized in the cytoplasmic membranes in the heterogenous host *E. coli* KNabc (Fig. 4). The sole co-expression of *umpA* and *umpB* could confer *E. coli* KNabc the capability of growing under halo-alkaline conditions (Fig. 3), which suggests that UmpA and UmpB may function as a hetero-dimer. That was established by the result that UmpB was co-immunoprecipitated by UmpA (Fig. 5B and C). Na^+^(Li^+^)/H^+^ antiport assays confirm that UmpAB is likely to function as a pH-dependent Na^+^(Li^+^)/H^+^ antiporter (Figs 7A,B and 8). Radchenko *et al*. showed that one mutant *E. coli* strain TO114 (the different designation with the same genotype as KNabc) lacking three major Na^+^(Li^+^)/H^+^ antiporters (NhaA, NhaB and ChaA) may be used for the identification of K^+^/H^+^ antiporter candidate genes^33^. K^+^/H^+^ antiport assay by using Na-free KCl reveals that UmpAB is also likely to function as a pH-dependent K^+^/H^+^ antiporter (Figs 7C and 8). Therefore, we propose that UmpAB should function as a two-component pH-dependent Na^+^(Li^+^, K^+^)/H^+^ antiporter.

Known Na^+^/H^+^ antiporters belonging to CPA family include two major sorts: single-gene Na^+^/H^+^ antiporters such as NhaA, NhaB, etc.^5, 8–21^ and multi-gene ones such as Mrp, Mnh, Pha or Sha^23–31^. In addition to two above-mentioned major categories, such proteins as ChaA, MleN, TetA(L), MdfA and Nap were also continually shown to exhibit Na^+^/H^+^ antiport activity^32–38^. However, a careful protein alignment at the NCBI website^43^ showed that there is no identity between either of UmpA or UmpB and all known specific Na^+^(Li^+^)/H^+^ antiporters, proteins with Na^+^(Li^+^)/H^+^ antiport activity, or even any subunit of multi-gene Na^+^/H^+^ antiporters. That was confirmed by the phylogenetic relationship that UmpA and UmpB are significantly distant with all known Na^+^/H^+^ antiporters and proteins with Na^+^/H^+^ antiport activity (Fig. 6). Also, UmpAB overlaps with each other (Fig. 2A) and co-expression of UmpAB could confer a halo-alkaline resistance phenotype (Fig. 3), which is similar to the report that PsmrAB, the sole two-component Na^+^/H^+^ antiporter, were characterized to be located in pairs^39^. However, PsmrA and PsmrB consist of three TMSs with 114 and 104 residues, respectively^39^. By comparison, UmpAB contain 254 and 271 residues, respectively, either of which is composed of seven putative TMSs (Fig. 2). Also, UmpAB showed K^+^/H^+^ antiport activity but PsmrAB not. The sole consistence is that one of the protein pair is longer than the other one and there is the higher identity between protein pairs. Even thus, UmpAB were also aligned with PsmrAB, but there is no identity between them. Therefore, UmpAB should encode a novel two-component Na^+^(Li^+^, K^+^)/H^+^ antiporter, which is significantly different from PsmrAB.

It’s very interesting that the sole UmpB exactly offered *E. coli* KNabc the better growth below 0.2 M NaCl, especially at 150 mM (Fig. 3B, Fig. S1) and a significant alkaline pH resistance at 7.5, but not at 8.0 (Fig. S1). Also, the sole expression of *umpA* offered *E. coli* KNabc a certain alkaline resistance at pH 7.5 in the presence of 50 mM NaCl (Fig. 3C, Fig. S1B), but inhibited the growth of *E. coli* KNabc even at pH 7.0 in the presence of 100 mM NaCl (Fig. 3B, Fig. S1C). That reveals that the sole expression of *umpA* or *umpB* could not be sufficient for the tolerance to 0.2 M NaCl and above, or alkaline pH resistance at 8.0, or even resulted in the inhibition of the growth. We speculate that the entire function of UmpAB as a Na^+^/H^+^ antiporter requires the formation of a hetero-dimer by UmpAB. Therefore, UmpA or UmpB could only offer limited NaCl or alkaline pH resistance, although either of them may form an unstable homo-dimer. Also, a significant difference is that the longer UmpB contains a hydrophilic C terminus in contrast to the shorter UmpA (Fig. 2). That suggests that the hydrophilic C terminus of UmpB may be quite important for the entire function of UmpAB as a Na^+^/H^+^ antiporter, since the sole UmpB exhibits the better NaCl tolerance and higher alkaline pH resistance, in comparison with the sole UmpA (Fig. 3B, Fig. S1). Also, UmpA contains a longer hydrophilic loop (Loop III-IV) between TMS III and TMS IV whereas UmpB contains a longer hydrophilic loop (Loop II-III) between TMS II and TMS III (Fig. 2). With the exception of several conserved residues, a majority of residues vary significantly in Loop III-IV of UmpA and Loop II-III of UmpB, but the similarity is that these two loops contain the rich charged or polar residues (Fig. 2). The hydrophobility and length of those two non-conserved longer loops may be essential for the entire function of UmpAB as a Na^+^/H^+^ antiporter. In the future study, we plan to modify UmpB by inserting Loop III-IV of UmpA into the corresponding position of UmpB or deleting the hydrophilic C terminus of UmpB, and also modify UmpA by inserting Loop II-III of UmpB into the corresponding position of UmpA and adding the hydrophilic C terminus of UmpB in order to test whether the sole modified UmpA or UmpB as a homo-dimer can exhibit the entire function of UmpAB as a Na^+^/H^+^ antiporter. Moreover, it should be reasonable that 36 hydrophobic residues were found to commonly exist between UmpA homologs and UmpB ones (Fig. 2B and C), since UmpAB were identified to be membrane proteins exhibiting Na^+^(Li^+^, K^+^)/H^+^ antiport activity (Figs 3–9). It should be pointed out that six charged residues and five polar ones, especially three acidic ones, are highly-conserved even when UmpAB homologs were broaden to a wider range of identities of 30–93% for UmpA and 28–90% for UmpB. In the future study, we also plan to replace the conserved charged or polar residues through alanine-scanning mutagenesis to identify whether they are involved in the Na^+^(Li^+^, K^+^)/H^+^ antiport activity of UmpAB. DUF1538 family represents a category of unknown membrane proteins collected in the Pfam database containing a domain of unknown function with No. 1538 designated DUF 1538 including several conserved glycine and phenylalanine residues^44^, which correspond to the sequences from No. 21 residue to No. 245 residue of UmpA and the ones from No. 17 residue to No. 240 residue of UmpB, respectively. As shown at the website https://www.ncbi.nlm.nih.gov/Structure/cdd/cddsrv.cgi?ascbin=8&maxaln=10&seltype=2&uid=pfam07556, no information suggested the exact function of DUF1538 family proteins. Since UmpAB clustered with all their homologs at a wide range of identities of 30–93% for UmpA and 28–90% for UmpB (Table S1) belonging to DUF1538 family with the bootstrap value of 98% (Fig. 6). Therefore, the function of UmpAB may represent those of this family proteins. In this study, we at least showed that UmpAB as a hetero-dimer exhibit Na^+^(Li^+^, K^+^)/H^+^ antiport activity, which is the first report on the functional analysis of a pair of proteins with unknown function as a representative of DUF1538 family protein pairs. The results presented in this manuscript trigger the understanding of the function of DUF1538 proteins and broaden the knowledge of novel Na^+^/H^+^ antiporters, especially for the ones from the moderately halophilic and alkaliphilic bacterium.

## Material and Methods

### Strains, plasmids and growth conditions

The strains and plasmids used in this study were listed in Table S2. *H. zhaodongensis* NEAU-ST10-25^T^ was incubated in 3% NaCl (optimum)-modified Sehgal-Gibbons (S-G) medium containing 1.0% tryptone, 0.5% yeast extract, 0.5% casein, 0.2% KCl, 0.3% sodium citrate, 2.0% MgSO_4_·7H_2_O add 3.0% NaCl at 35 °C and pH 7.2-7.4^40^. The Na^+^/H^+^ antiporter-deficient strain of *E. coli* KNabc (*nhaA*::Km^R^, *nhaB*::Em^R^, *chaA*::Cm^R^)^7^ and its transformant cells were grown aerobically in the KCl-modified Luria-Bertani (LBK) medium consisting of 1.0% tryptone, 0.5% yeast extract and 87 mM KCl at 37 °C as previously described by Karpel *et al*.^5^, to which NaCl or LiCl was added at indicated concentrations when necessary. Ampicillin was added to a final concentration of 50 μg∙ml^−1^ for the selection and growth of transformants. The pre-cultures of *E. coli* KNabc transformant cells was prepared in LBK medium, followed by incubation overnight at 37 °C. For the salt tolerance test, 1% of the overnight cultures were inoculated into the 5-ml fresh LBK medium at pH 7.0, to which NaCl (0–0.6 M) or LiCl (0–40 mM) was added at indicated concentrations, followed by incubation at 37 °C. To test the effect of pH on cell growth, 1% of the overnight cultures were inoculated into the 5-ml fresh LBK medium containing 50 mM NaCl at indicated pH values (7.0–8.5) by adding the Hepes-Tris buffer (final concentration at 100 mM), followed by incubation at 37 °C. It should be pointed out that Na^+^(Li^+^)/H^+^ antiporters can offer alkaline pH resistance only in the presence of Na^+^ or Li^+^. Therefore, a certain amount of Na^+^ such as 50 mM NaCl needs to be added to the tested medium^1–4^. In order to further test the respective NaCl or alkaline pH resistance of either UmpA or UmpB, 1% of the overnight cultures were inoculated into the 5-ml fresh LBK medium at NaCl concentrations varied 0 to 150 mM at the pH values from 7.0 to 8.5, followed by incubation at 37 °C. The above-mentioned cell growth was ended after 24 h and monitored turbidimetrically at 600 nm. Electrocompetent *E. coli* cells were prepared and electroporated according to the protocol described in our previous study^39^.

### Screening of the Na^+^(Li^+^)/H^+^ antiporter gene

The genomic DNA was extracted from strain NEAU-ST10-25^T^ and partially digested with *Sau*3AI. The DNA fragments with 4–10 kb were separated by agarose electrophoresis and ligated into pUC18, which had been digested with *Bam*HI and dephosphorylated with bacterial alkaline phosphatase, using a T4 DNA ligase. Electrocompetent cells of *E. coli* KNabc were transformed with the ligated reaction mixture and spread on LBK medium plates containing 0.2 M NaCl, 1.5% agar and 50 mg∙ml^−1^ of ampicillin. The plates were incubated at 37 °C for 20 h and colonies picked for further studies. Subcloning of each of ORF2-4 or ORF3-4 including their respective promoter-like and SD sequences was carried out by PCR amplification, purification and re-ligation into pUC18 vector as shown in Fig. 1. For the subcloning of 5′-end truncated ORF1, it was fused in frame with an N-terminal His_6_ tag in an expression vector, pET19 (Novagen Ltd., USA). To confirm whether co-expression of ORF3 and ORF4, or the sole expression of either one could rescue *E. coli* KNabc, ORF3 was fused in frame with an N-terminal His_6_ tag in the multiple cloning site 1 of a co-expression vector, pETDuet-1 (Novagen Ltd., USA), and/or ORF4 followed by an in-frame c-Myc tag and a stop codon, the sequences of which were designed in the reverse primer R4 for ORF4, was inserted into the multiple cloning site 2 in the same plasmid through PCR amplification, restriction enzyme digestion and ligation. The sequences of primers used in this study were listed in Table S2.

### Preparation of everted membrane vesicles

*E. coli* KNabc cells carrying pUC-umpAB and pUC18 (as a negative control) were grown in LBK medium up to the mid-exponential phase and harvested by centrifugation at 5000 g, 4 °C for 10 min. Everted membrane vesicles were prepared by breaking cells with a JG-1A French pressure cell press (NingBo Scientz Biotechnology Co., Ltd, China) at 2, 000 psi and collected by ultracentrifugation at 100, 000 g for 1 h as previously described by Rosen^45^. Everted membrane vesicles were re-suspended in the 10 mM Tris-HCl (pH 7.5) buffer containing 140 mM choline chloride, 0.5 mM dithiothreitol (DTT), 250 mM sucrose, a protease inhibitor tablet (Roche) and 1 mM phenylmethylsulfonyl fluoride (PMSF) and then stored at −80 °C.

### Preparation of cell extract, membrane fraction and cytoplasmic fraction

*E. coli* KNabc cells carrying pETDuet-1-umpAB and the empty vector pETDuet-1 (as a negative control) were grown in the LBK medium to OD_600 nm_ between 0.4 and 0.6 at 37 °C, followed by induction by the addition of isopropyl-β-D-thiogalactoside to a final concentration of 1 mM at 28 °C for an additional 6 h and then harvested by centrifugation at 5, 000 g, 4 °C for 10 min and washed three times with 10 mM Tris-HCl (pH 7.5). Cell pellets were frozen at −80 °C overnight to weaken the cell wall and re-suspended in an ice-cold lysis buffer containing 50 mM Tris-Cl (pH 8.0), 2 mM EDTA, 100 mM NaCl and 0.1% Triton X-100. Cell suspension was lysed in the above-mentioned ice-cold lysis buffer plus 1 mM PMSF, 1 mM DTT and 100 μg∙ml^−1^ lysozyme via an JY92-IIN ultrasonic cell mixer (NingBo Scientz Biotechnology Co., Ltd, China) in a pulsed mode (cycles: 2 sec ON followed by 3 sec OFF) until the lysate changed from an opaque solution into a less turbid solution. The lysed sample was centrifuged at 5, 000 g at 4 °C for 10 min to remove large debris fragments and unlysed cells. A part of supernatant was sampled as a representative of the cell extract including membrane and cytoplasmic fractions and the remain supernatant was ultracentrifuged at 100, 000 g for 1 h as described by Rosen^45^ to separate membrane fraction (pellets) from cytoplasmic one (supernatant). The samples as the respective representative of cell extract, membrane fraction and cytoplasmic fraction were used for the SDS-PAGE and western blot analysis.

### Co-immunoprecipitation

The 30-μl above-prepared membrane fraction containing 40 μg∙μl^−1^ total membrane protein from *E. coli* KNabc cells carrying pETDuet-1-umpAB stored in the 10 mM Tris-HCl (pH 7.5) buffer containing 140 mM choline chloride, 0.5 mM DTT, 250 mM sucrose, a protease inhibitor tablet (Roche Ltd., China) and 1 mM PMSF were re-suspended in 400 μl of an ice-cold commercially-available buffer (Beyotime Biotechnology Co. Ltd, China) containing 20 mM Tris-HCl (pH 7.5), 150 mM NaCl, 1% Triton X-100 and a certain amount of protease inhibitors including sodium pyrophosphate, β-glycerophosphate, EDTA, sodium ortovanadate and leupeptin. And then the primary antibody [a rabbit anti-His_6_-tag antibody (Abcom Ltd, China) for UmpA] was added to the membrane fraction and incubated with rotation overnight at 4 °C. 50% slurry of protein agarose A-sepharose (Beyotime Biotechnology Co. Ltd, China) was then added and the incubation continued for an additional 4 h. After washing five times with the ice-cold above-mentioned commercially-available buffer, the precipitates, together with membrane fraction (as a positive control) and the rabbit IgG mixed with the solubilized membrane proteins by the protein agarose A-sepharose (as a negative control), were used for the SDS-PAGE and western blot analysis.

### SDS-PAGE and western blots

SDS-PAGE and western blots were performed as described by Green *et al*.^46^. The samples as the respective representatives of cell extract, membrane fraction and cytoplasmic fraction were used for the detection and localization of UmpA and UmpB by western blot. His_6_-tag detection was done by using a rabbit anti-His_6_-tag antibody (Abcom Ltd, China) and a goat anti-rabbit horseradish peroxidase-labelled secondary antibody (Nachuan Biotechnology Co., Ltd, Changchun, China). c-Myc-tag detection was done by using a mouse anti-c-Myc-tag antibody (Abcom Ltd, China) and a goat anti-mouse horseradish peroxidase-labelled secondary antibody (Nachuan Biotechnology Co., Ltd, Changchun, China). The precipitates from co-immunoprecipitation, together with membrane fraction (as a positive control) and the rabbit IgG mixed with the solubilized membrane proteins by the protein agarose A-sepharose (as a negative control), were used for the determination of UmpA and UmpB as a hetero-dimer by western blot. To avoid the visualization of the light chain and the heavy chain from a rabbit anti-His_6_-tag antibody in the precipitates, His_6_-tag detection was done by using a mouse anti-His_6_-tag antibody (Abcom Ltd, China) and a goat anti-mouse horseradish peroxidase-labelled secondary antibody (Nachuan Biotechnology Co., Ltd, Changchun, China). c-Myc-tag detection was done by using the same primary antibody and secondary antibody as described above. The BeyoECL Star kit (Beyotime Biotechnology Co. Ltd, China) was used and antibody binding was visualized by a Tanon-5200 multi chemiluminescence imaging system (Tanon Co. Ltd, China).

### Assays of Na^+^(Li^+^, K^+^)/H^+^ antiport activity

Na^+^(Li^+^, K^+^)/H^+^ antiport activity of everted membrane vesicles was estimated according to the extent of the collapse of a transmembrane proton gradient, with acridine orange as the pH indicator, as described by Rosen^45^. The assay mixture contained 10 mM BTP (BisTris-Propane) (at the indicated pH from 6.5 to 9.5), 140 mM choline chloride, 5 mM MgSO_4_, 2 μM acridine orange and membrane vesicles (equivalent of 40 μg total membrane protein). Tris-D-lactic acid (final concentration at 5 mM) was added to initiate respiration. After the fluorescence quenching reached steady state, NaCl, LiCl or Na-free KCl with high purity (99.9995%, Sigma-Aldrich Co. LLC.) (To avoid the contamination of traces of NaCl) was added to the final concentration of 5 mM and then the fluorescence was dequenched. The ratio of fluorescence dequenching extent by NaCl, LiCl or Na-free KCl to the fluorescence quenching one by Tris-D-lactic acid was recorded as a representative of Na^+^(Li^+^, K^+^)/H^+^ antiport activity. Measurements were conducted using a Hitachi F-7000 fluorescence spectrophotometer (Hitachi Ltd, Tokyo, Japan) with excitation at 492 nm (10-mm slit) and emission at 526 nm (10-mm slit), respectively.

### Calculation of K_0.5_ values of UmpAB for the cations

For the calculation of K_0.5_ values of UmpAB for the cations, pH was adjusted to 9.0 based on the highest Na^+^(Li^+^, K^+^)/H^+^ antiport activity, and the different Na^+^, Li^+^ or K^+^ concentrations were varied from 0 to 10 mM. Fluorescence dequenching percentages at the corresponding cation concentrations were recorded as their respective representatives of Na^+^(Li^+^, K^+^)/H^+^ antiport activity and then plotted as the respective functions of the corresponding cation concentrations. Finally, K_0.5_ values of UmpAB for Na^+^, Li^+^ and K^+^ were obtained, respectively, by fitting a hyperbolic function to the data with OriginPro 8.6.

### DNA manipulation and bioinformatics analyses

Preparation of plasmid DNA, extraction of genomic DNA, restriction enzyme digestion and ligation were carried out as described by Green *et al*.^46^. DNA sequencing was performed by Beijing Genomics Institute (Beijing, China). The analyses for ORF, hydrophobicity and amino acid composition were carried out with the DNAMAN 6.0 software. Protein sequence alignment was performed through the National Center for Biotechnology Information (NCBI) using the website https://blast.ncbi.nlm.nih.gov/Blast.cgi?PROGRAM=blastp&PAGE_TYPE=BlastSearch&LINK_LOC=blasthome^43^. For the construction of phylogenetic tree, the multiple alignments of the selected protein sequences were run by using the ClustalX program^47^. The phylogenetic tree was constructed by using MEGA 5.0 using the neighbour-joining method^48^. The stability of clusters was ascertained by performing a bootstrap analysis (1000 replications). Promoter prediction was performed by using the website http://www.fruitfly.org/seq_tools/promoter.html. Prediction of the transmembrane segments was done by submitting the deduced amino acid sequences of UmpA or UmpB to the website http://www.tcdb.org/progs/TMS.php ^49^.

### Protein content determination

Protein content in everted membrane vesicles was determined by the method of Lowry *et al*.^50^ with bovine serum albumin as a standard.

### Nucleotide sequence accession number

The nucleotide sequence reported in this study has been submitted to GenBank database with the accession number KY241440.

## Electronic supplementary material

Supplemental Information
