# Supplementary material for: Characterization of a novel two-component Na+(Li+, K+)/H+ antiporter from Halomonas zhaodongensis
Source: Sci Rep. 2017 Jun 26;7:4221. doi: 10.1038/s41598-017-04236-0 (PMC5484666; doi:10.1038/s41598-017-04236-0)
Supplement: Supplementary file 1 — Supplemental Information [file 41598_2017_4236_MOESM1_ESM.pdf]

Supplemental information

Characterization of a novel two-component $Na^+(Li^+, K^+)/H^+$ antiporter from *Halomonas zhaodongensis*

Lin Meng, Fankui Meng, Rui Zhang, Zhenglai Zhang, Ping Dong, Kaifu Sun, Jin Chen, Wei Zhang, Mingxue Yan, Jincheng Li, Heba Abdel-motaal and Juquan Jiang*

Department of Microbiology and Biotechnology, College of Life Sciences, Northeast Agricultural University, Harbin, PR China

*Authors for correspondence: Juquan Jiang, Tel: 86-451-55191132. Fax: 86-451-55190413. E-mail address: jjqdainty@163.com

Table S1.The identities of UmpAB from *Halomonas zhaodongensis* with the selected homologs.

Protein ID	Organism name	Accession.version No.	No. of amino acid residues	Identity (%) with Hz_UmpA	Identity (%) with Hz_UmpB
Ha_UmpA	*Halomonas alkaliantarctica*	WP_030072713.1	254	93	42
Ha_UmpB		WP_030072711.1	271	44	90
Hs_UmpA	*Halomonas stevensii*	WP_016913696.1	255	87	44
Hs_UmpB		WP_016913695.1	262	42	87
Hsh_UmpA	*Halomonas* sp. HL-93	SBR49102.1	255	84	42
Hsh_UmpB		SBR49103.1	268	43	80
Hsb_UmpA	*Halomonas* sp. BC04	WP_043518463.1	250	74	38
Hsb_UmpB		WP_043518462.1	257	42	76
Th_UmpA	*Thiohalospira halophila*	SFD15376.1	244	70	41
Th_UmpB		SFD15402.1	258	41	57
Ve_UmpA	*Vibrio ezurae*	WP_021712002.1	241	65	39
Ve_UmpB		WP_021712001.1	265	44	59
Cs_UmpA	*Candidatus* Scalindua sp. BSI-1	ODS32921.1	245	59	43
Cs_UmpB		ODS32922.1	267	47	52
Vx_UmpA	*Vibrio xiamenensis*	SDH66041.1	245	59	38
Vx_UmpB		SDH66014.1	261	44	56
Dp_UmpA	*Desulfobacula phenolica*	SDU55681.1	244	57	45
Dp_UmpB		SDU55692.1	264	45	57
Ml_UmpA	*Methylosarcina lacus*	WP_024299360.1	242	57	39
Ml_UmpB		WP_024299361.1	259	41	52
Mf_UmpA	*Mariprofundus ferrooxydans*	WP_009850723.1	249	56	43
Mf_UmpB		WP_009850724.1	255	45	55
Mm_UmpA	*Methyloglobulus morosus*	WP_023494111.1	244	54	38
Mm_UmpB		WP_023494112.1	253	43	58
Bc_UmpA	*Bacillus campisalis*	WP_046525099.1	229	40	37
Bc_UmpB		WP_046525098.1	243	34	33
Bo_UmpA	*Bacillus oryziterrae*	WP_017754140.1	230	38	35
Bo_UmpB		WP_017754141.1	249	32	30
Pd_UmpA	*Planococcus donghaensis*	ANU22115.1	231	34	32
Pd_UmpB		ANU22114.1	242	36	32
Bm_UmpA	*Bacillus marmarensis*	WP_022629473.1	230	35	34
Bm_UmpB		WP_022629472.1	249	36	31
Pp_UmpA	*Planococcus plakortidis*	ANU20467.1	231	36	32
Pp_UmpB		ANU20468.1	247	35	30
Ea_UmpA	*Enterococcus asini*	WP_010753786.1	227	30	29
Ea_UmpB		WP_010753785.1	258	30	28

Table S2. The strains, plasmids and primers used in this study.

Strains, plasmids or primers	Relevant phenotype, genotype or primer sequence	The source
Strains		
Halomonas zhaodongensis NEAU-ST10-39^{T}	type strain of *Halomonas zhaodongensis*, a moderate halophile and alkaliphile	Isolated and identified by our group[40]
Escherichia coli KNabc	*nhaA*::Km^{R}, *nhaB*::Em^{R}, *chaA*::Cm^{R}	Donated by Professor Terry A. Krulwich[7]
Plasmids		
pUC18	Cloning vector	Takara Biotechnology (Dalian) Co.,Ltd., China
pUC-ZD-6	pUC18 carrying a 4.2-kb DNA fragment with	This study
pUC-umpA	pUC18 carrying the sole *umpA* gene	This study
pUC-umpB	pUC18 carrying the sole *umpB* gene	This study
pUC-umpAB	pUC18 carrying *umpA* and *umpB* genes	This study
pUC-ORF2	pUC18 carrying the sole ORF2	This study
pET19	Over-expression vector	Novagen Ltd., USA
pET19-truncated ORF1	pET19 carrying 5'-end truncated ORF1	This study
pETDuet-1	Co-expression vector for two target genes	Novagen Ltd., USA
pETDuet-1-umpA	pETDuet-1 carrying the sole *umpA* gene	This study
pETDuet-1-umpB	pETDuet-1 carrying the sole *umpB* gene	This study
pETDuet-1-umpAB	pETDuet-1 carrying *umpA* and *umpB* genes	This study
Primers		
F1, the forward primer for *umpA* in pUC-umpA or *umpAB* in pUC-umpAB	5'-GAATTCGATTGTGATGTTTATTGCG-3' (*Eco*RI, underlined)	This study
F2, the forward primer for *umpA* in pUC-umpB	5'-GAATTCCTGGGGAAGTCGTATCAG-3' (*Eco*RI, underlined)	This study
F3, the forward primer for *umpA* in pETDuet-1-umpA or pETDuet-1-umpAB	5'-GAATTCGATGAATATAGTTTATTCG-3' (*Eco*RI, underlined)	This study
F4, the forward primer for *umpB* in pETDuet-1-umpB or pETDuet-1-umpAB	5'-CATATGATATTACTCACGATTTTC-3' (*Nde*I, underlined)	This study
F5, the forward primer for 5'-end truncated ORF1 in pET19-truncated ORF1	5'-CATATGGTCCCTGCCTCTATTGCGCT-3' (*Eco*RI, underlined)	This study
F6, the forward primer for ORF2 in pUC-ORF2	5'-GAATTCTTTCGATTGCCCTGCAAACC-3' (*Eco*RI, underlined)	This study
R1, the reverse primer for *umpA* in pUC-umpA	5'-AAGCTTTTAGGTGCTTCCCGTCAC-3' (*Hin*dIII, underlined)	This study
R2, the reverse primer for *umpB* in pUC-umpB or *umpAB* in pUC-umpAB	5'-AAGCTTGCTTGTAGAATGTCGTCG-3' (*Hin*dIII, underlined)	This study
R3, the reverse primer for *umpA* in pETDuet-1-umpA or pETDuet-1-umpAB	5'-AAGCTTTTAGGTGCTTCCCG-3' (*Hin*dIII, underlined)	This study
R4, the reverse primer for *umpB* in pETDuet-1-umpB or pETDuet-1-umpAB	5'-GGTACCTTACAGGTCTTCTTCAGAGATCAGTTTCTGTTCTGGTCTAAGCGCATTATTATCTCCTT-3' (*Kpn*I, underlined; *myc*-tag, double-underlined)	This study
R5, the reverse primer for 5'-end truncated ORF1 in pET19-truncated ORF1	5'-GGATCCTTAGAAGCTGCCAAACAGAG-3' (*Bam*HI, underlined)	This study
R6, the reverse primer for ORF2 in pUC-ORF2	5'-GGATCCGTGCTATCCAGCGTGTGCCA-3' (*Bam*HI, underlined)	This study

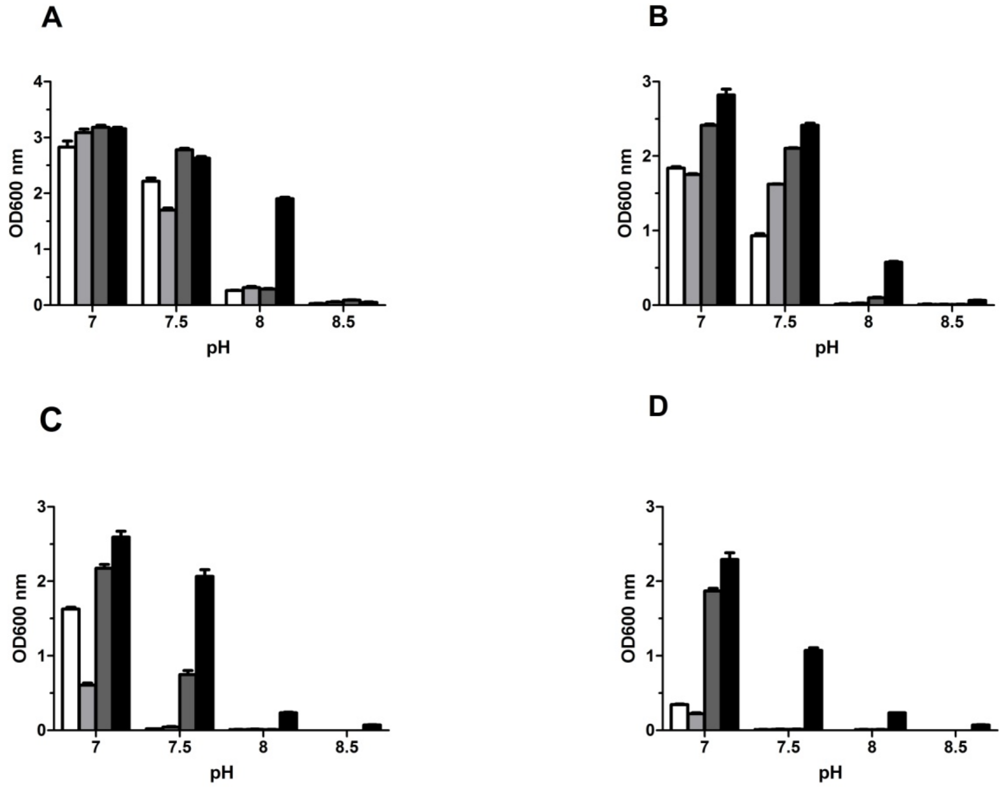

Fig. S1. Growth test for NaCl tolerance or alkaline pH resistance of *E. coli* KNabc by UmpA, UmpB or both.

In order to further test NaCl tolerance or alkaline pH resistance of either UmpA or UmpB, 1 % of the overnight cultures of *E. coli* KNabc/pUC-umpAB (black column), KNabc/pUC-umpA (lightgrey column), KNabc/pUC-umpB (grey column) and KNabc/pUC18 (white column) were grown, respectively, in the LBK medium containing NaCl concentrations of 0 (A), 50 (B), 100 (C) and 150 mM (D) at the pH values from 7.0 to 8.5. The above-mentioned cell growth was ended after 24 h and monitored turbidimetrically at 600 nm. Each data point represents the average of three independent determinations.
